# Systematic Approach to Address Early Pandemic's Diagnostic Unmet Needs

**DOI:** 10.3389/fmicb.2022.910156

**Published:** 2022-06-17

**Authors:** Catherine Cabrera, Kanoelani Pilobello, Steven Dalvin, Johanna Bobrow, Darshi Shah, Lori Freed Garg, Sujata Chalise, Patrick Doyle, Glenn A. Miller, David R. Walt, Sara Suliman, Pawan Jolly

**Affiliations:** ^1^Massachusetts Institute of Technology Lincoln Laboratory, Lexington, MA, United States; ^2^Wyss Institute for Biologically Inspired Engineering, Harvard University, Boston, MA, United States; ^3^Department of Biomedical Engineering, Boston University, Boston, MA, United States; ^4^Global Health Innovation Lab, Department of Emergency Medicine, Massachusetts General Hospital, Boston, MA, United States; ^5^Department of Pathology, Brigham and Women's Hospital, Harvard Medical School, Boston, MA, United States; ^6^Brigham and Women's Hospital, Boston, MA, United States; ^7^Mass General Brigham Incorporated, Boston, MA, United States; ^8^Harvard Medical School, Boston, MA, United States; ^9^Division of Rheumatology, Inflammation and Immunity, Brigham and Women's Hospital, Boston, MA, United States; ^10^Mass General Brigham Center for COVID Innovation, Boston, MA, United States; ^11^Zuckerberg San Francisco General Hospital, Division of Experimental Medicine, University of California, San Francisco, San Francisco, CA, United States

**Keywords:** COVID-19, point-of-service, diagnostic, SARS-CoV-2, rubric system

## Abstract

During the first few months of the global Severe Acute Respiratory Syndrome Coronavirus-2 (SARS-CoV-2) pandemic, the medical research community had to expeditiously develop, select, and deploy novel diagnostic methods and tools to address the numerous testing challenges presented by the novel virus. Integrating a systematic approach to diagnostic selection with a rapid validation protocol in a clinical setting can shorten the timeline to bring new technologies to practice. In response to the urgent need to provide tools for identifying SARS-CoV-2-positive individuals, we developed a framework for assessing technologies against a set of prioritized performance metrics to guide device selection. We also developed and proposed clinical validation frameworks for the rapid screening of new technologies. The rubric described here represents a versatile approach that can be extended to future technology assessments and can be implemented in preparation for future emerging pathogens.

## Introduction

The novel coronavirus, now designated Severe Acute Respiratory Syndrome Coronavirus-2 (SARS-CoV-2), responsible for the Coronavirus Disease of 2019 (COVID-19), has reportedly infected over 416 million people as of February 17, 2022 (Dong et al., 2020; Worldometer, 2021). The need to effectively triage patients, inform treatment decisions, perform contact tracing to control infectious outbreaks, and collect epidemiological data about infection spread to inform national and state-level policies have highlighted the critical importance of diagnostic testing (Binnicker, 2020). Early in the pandemic, the need for diagnostic testing was quickly recognized in resource-constrained healthcare settings having limited hospital staff, personal protective equipment (PPE) shortages, and insufficient negative pressure rooms (Ferretti et al., 2020). The ability to accurately triage SARS-CoV-2-infected patients with testing was essential to protecting healthcare workers and patients alike. In countries with extensive contact-tracing programs such as South Korea, high volume testing paired with quarantine efforts was found to dramatically slow viral spread (Shim et al., 2020; Wrighton and Lawrence, 2020). Finally, as countries pushed to reopen their economies, it became evident that diagnostic testing would be critical not only for mass scale asymptomatic testing to enable institutions to resume operating, but also for generating the epidemiological data to closely monitor the spread of infection and inform decisions around closing and reopening businesses (Cheng et al., 2020).

As the need for diagnostics grew, the challenges and uncertainties associated with obtaining such diagnostics emerged. Some of these challenges were rooted in deconstructing the biological mechanisms mediating susceptibility to infection and disease, such as a lack of understanding of tissue and cell-specific compartmentalization during different phases of infection (Bourgonje et al., 2020; Hoffmann et al., 2020), the time course of infection and infectivity, and the nature and time course of the immune response to the virus. Other challenges were more logistical or operational; for example, what pre-existing diagnostic systems could be adapted or repurposed to detect SARS-CoV-2 to confirm COVID-19 diagnosis, what pre-existing supply chains could be leveraged or redirected in support of this effort, what sensitivity and specificity levels were required, and what infrastructure and personnel support were required and available in different kinds of locations (Frisch et al., 2021). Of particular concern was the lack of options for point-of-care/point-of-need environments, areas where trained staff and time are limited (relative to centralized laboratories), but demand was (and remains) very high.

While the critical role of diagnostics has been made clear, testing capacity and turnaround time have been significant barriers to more effective testing strategies (Clipman et al., 2021). One of the major problems faced during the first months of the pandemic was the shortage of molecular testing assays in general (Ward et al., 2020) and the absence of diagnostics that were appropriate for point-of-care settings in particular; as the diagnostic devices that were initially available were both too large and too complex to be used in decentralized patient care settings (Giri et al., 2021). More importantly, numerous factors hindered the testing capacity even when the diagnostic devices were available. As noted in the analyst report by Mckinsey & Co. (Behnam et al., 2020), even when the diagnostic devices were available, there often was a shortage of sample collection supplies, required reagents, and qualified personnel to perform the tests. These supply chain challenges exacerbated the challenge posed by the inherent urgency of the need to identify infected individuals at point-of-care during a global pandemic caused by a novel pathogen.

This paper summarizes efforts developed by a diverse team of subject matter experts to rapidly address these uncertainties, provide actionable guidance to decision-makers, and create a framework that could be used to support similar analyses in the event of future pandemics. The scope of the effort was limited to early detection of COVID-19 and how to address challenges with limited clinical indicators to minimize the time to clinical validation of the diagnostic technology. The paper aims to address the following considerations: (1) Develop a framework for the broader diagnostics and healthcare provider communities to evaluate new testing methodologies and ease future technology assessment efforts; (2) Catalyze a discussion within this research community on how to prepare for the next emerging pathogen; and (3) Propose necessary clinical validation frameworks and lessons learned from this process to inform and improve subsequent analyses.

## Methods

### Horizon Scanning and Acquisition of Information

**Figure 1:**
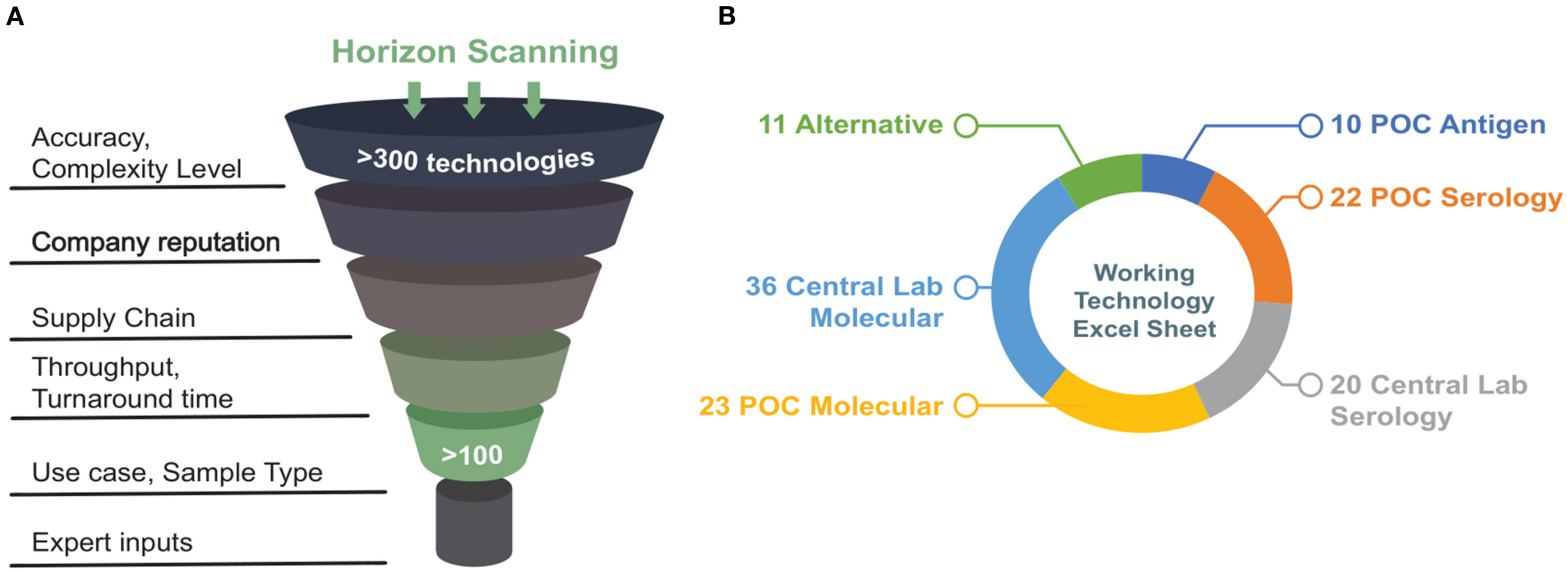
**(A)** Schematic demonstrating initial criteria used for horizon scanning. **(B)** Summary of technologies that met initial requirements after a first-pass scan grouped by sample type (figure adapted from https://covidinnovation.partners.org/point-of-service-urgent-care/).

A deep horizon scan of commercially available viral RNA and serology tests was performed as a first step. The results were stored in a database comprising technologies in different phases of development. The database was populated using the FDA's list of emergency use authorization (EUA)-approved and EUA-pending tests, diagnostics industry newsletters, press releases, and professional networks and online repositories. An example of one of those repositories is from the Foundation for Innovative New Diagnostics (FIND), a non-profit collaborating center of the World Health Organization (WHO). This database is an up-to-date resource of manufacturer-independent evaluation data gathered from many international laboratories for point-of-care molecular and rapid antigen tests for SARS-CoV-2, as well as serological tests to detect antibodies against SARS-CoV-2 (FIND, 2020). We developed a set of initial inclusion criteria based on sensitivity, specificity, and supply-chain logistics, formalized them into a questionnaire to consolidate information for initial assessment (Supplementary Figure 1). In addition to in-house evaluations from what became the Diagnostic Accelerator (DA) working group, these public evaluation results were used to guide the selection of test platforms. Figure 1A represents the initial criteria used for horizon scanning performed in April 2020.

### Early Assessment Criteria

A systems analysis approach (Delaney et al., 2015) was applied to assess emerging diagnostics that might be suitable for point-of-care use. The overall goal was to provide recommendations for technologies that could be acquired, evaluated, and ideally be deployed as quickly as possible to support diagnostic needs in a clinical setting. More specifically, the focus was to review and recommend diagnostics that could be used in Point-of-Care (POC) and/or urgent care settings, and that directly sensed the presence of SARS-CoV-2 through assays targeting viral RNA. Initially, a broader range of targets was considered, the most significant of which were viral protein antigens (Figure 1B; Supplementary Table 1). However, when this review was being conducted (April-June 2020), antigen- and serology-based assays were not mature enough to be deployed immediately and did not yet provide the same degree of confidence as molecular (RNA) assays. Therefore, this discussion will be focused exclusively on molecular assays targeting viral RNA.

**Figure 2:**
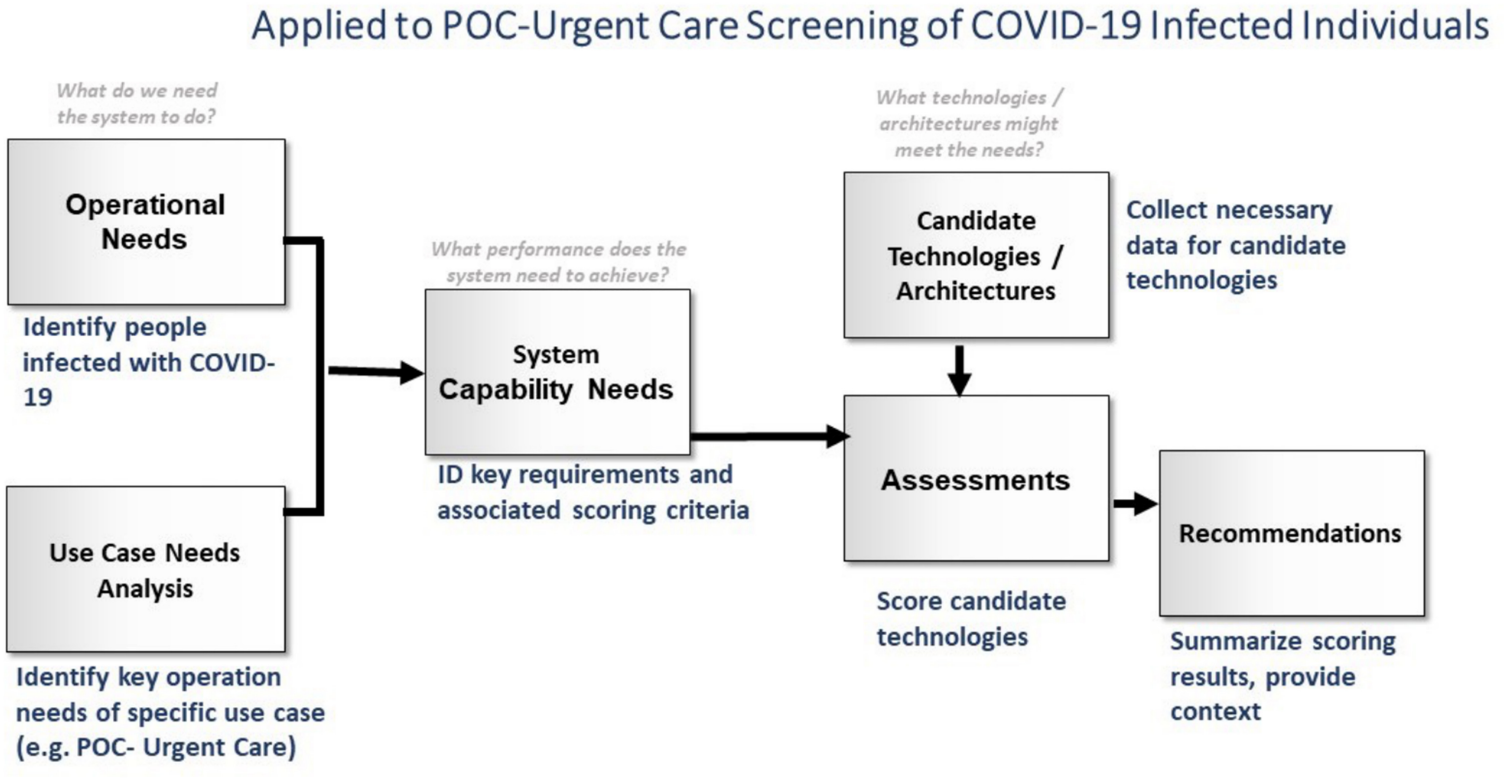
Overview of the technology assessment methodology (figure adapted from https://covidinnovation.partners.org/point-of-service-urgent-care/).

The analysis focused on assessing tests that could be performed in settings, such as point-of-care (POC) environments, with fewer resources than regional hospitals and central laboratories. Testing in these settings would reduce the centralized diagnostic burden and provide more immediate responses to medical care professionals. This diagnostics assessment effort had two goals. The first and most critical was to rapidly identify the most promising technologies to address the urgent needs to counter the COVID-19 pandemic through point-of-service diagnostics that provide timely and reliable information. The second goal was to develop a formalized way to structure, execute, and document this assessment process to inform the medical community (and others) by making this process transparent, comprehensible, and supportive of similar decision-making efforts in the future. A systems analysis-based approach, which is well-suited to identifying possible technical solutions to a challenging and complex problem, was adapted to these specific goals. While the process (Figure 2) is displayed linearly, feedback loops were developed between boxes to refine efforts and strengthen the final analysis. Experts in the clinical, industry, and research spheres, including hospital leadership, were consulted frequently to ensure the recommendations would suit the clinical need.

## Results

### Adapted Systems Analysis Approach

Systems analysis is an approach to understanding and addressing complex challenges (Delaney et al., 2015). It provides a framework for conceptualizing the assessment and decision-making process. Through a formalized, step-by-step methodology, a diverse team can reach consensus on the problems to be addressed and the solution options, rank/prioritize those options, and generate a set of consensus recommendations (Delaney et al., 2015). Formalizing and documenting this process enables the effective inclusion of new perspectives, data, and requirements, which enables the generation of updated recommendations in response to changing conditions. This formalized, documented process also provides a transparent roadmap to how recommendations were generated, which should, in theory, allow the broader community to easily understand the decision-making process and facilitate solicitation and incorporation of feedback from those community members.

Applying this systems analysis approach to the challenges of selecting point-of-care diagnostics for COVID-19, including supply chain constraints, required two parallel efforts—(1) Understanding and defining the operational need (in this case, enabling diagnosis of SARS-CoV-2 infection in POC settings) and (2) Determining which technical options are available to meet that need (in this case, diagnostics). These parallel efforts were brought together in an assessment phase, in which “what-is-needed” is compared to “what-is-possible”, and that evaluation informs the ultimate recommendations of technologies to pursue. Ideally, these parallel efforts are described with the same terminology, enabling a clear consideration of how candidate technologies meet operational goals. However, during the early pandemic response, information about disease pathogenesis and symptomology was unclear, new diagnostic technologies appeared daily, and supply chains quickly went from a topic rarely discussed to a vital part of all decision-making processes. Supply chain measures how quickly core reagents could be obtained to perform the diagnostic tests. Such reagents included pipette tips, polymerases, RNA extraction kits, specialized swabs and viral transport media, and other reagents. As a rule of thumb, we prioritized technologies where the tests could be obtained within a week. However, supply chain ebbed and flowed during different SARS-CoV-2 surges, and no single technology could fulfill the unmet need (Humble et al., 2021). Hence, we aimed to diversify the tests in evaluation, and predicted that technologies that had simpler workflows and required fewer specialized reagents to pose fewer supply chain obstacles, such as the Fluxergy CoVID-19 Sample-to-Answer RT-PCR (Rawlings et al., 2021). It became clear that the need to provide actionable information quickly precluded a complete, formal, and deliberate systems analysis. This process was supported by the redeployment of dozens of administrative and research staff throughout the Mass General Brigham (MGB) system and beyond to assist with screening and evaluation of new diagnostics as they were developed and brought to market. Key components of this process were retained as necessary to facilitate communication, optimize time spent researching technologies, and enable documentation of this fast-moving effort such that it could be readily revised as new information became available and could be leveraged by other groups facing similar challenges during this and future pandemics. Efforts were therefore focused on the aspects of the methodology that were most critical to the primary analysis—assessing diagnostic technologies for use in POS settings—and directed toward parallel creation of both an assessment rubric and a technology summary table. Updates and preliminary findings from each group were shared daily and used to guide the work of both groups.

### Operational and Use-Case Needs Analysis

The use case motivating this assessment was detecting SARS-CoV-2 infections in individuals at the point-of-care, to inform medical and public health decisions (e.g., further treatment, isolation, and patient triage). Other use cases, such as population-level surveillance, travel, and return-to-work consideration, were outside the scope of our efforts. To achieve the goal of determining which diagnostic technologies were best-suited for use in POC settings, both terminology and scope had to be defined. It became clear that the working group members had varying definitions of “point-of-care” and “diagnostic”. It was found that working within the systems analysis framework, which provides a formalized process and tools for defining key terms, allowed the team to both reach consensus and clearly document our process and terms.

**Figure 3:**
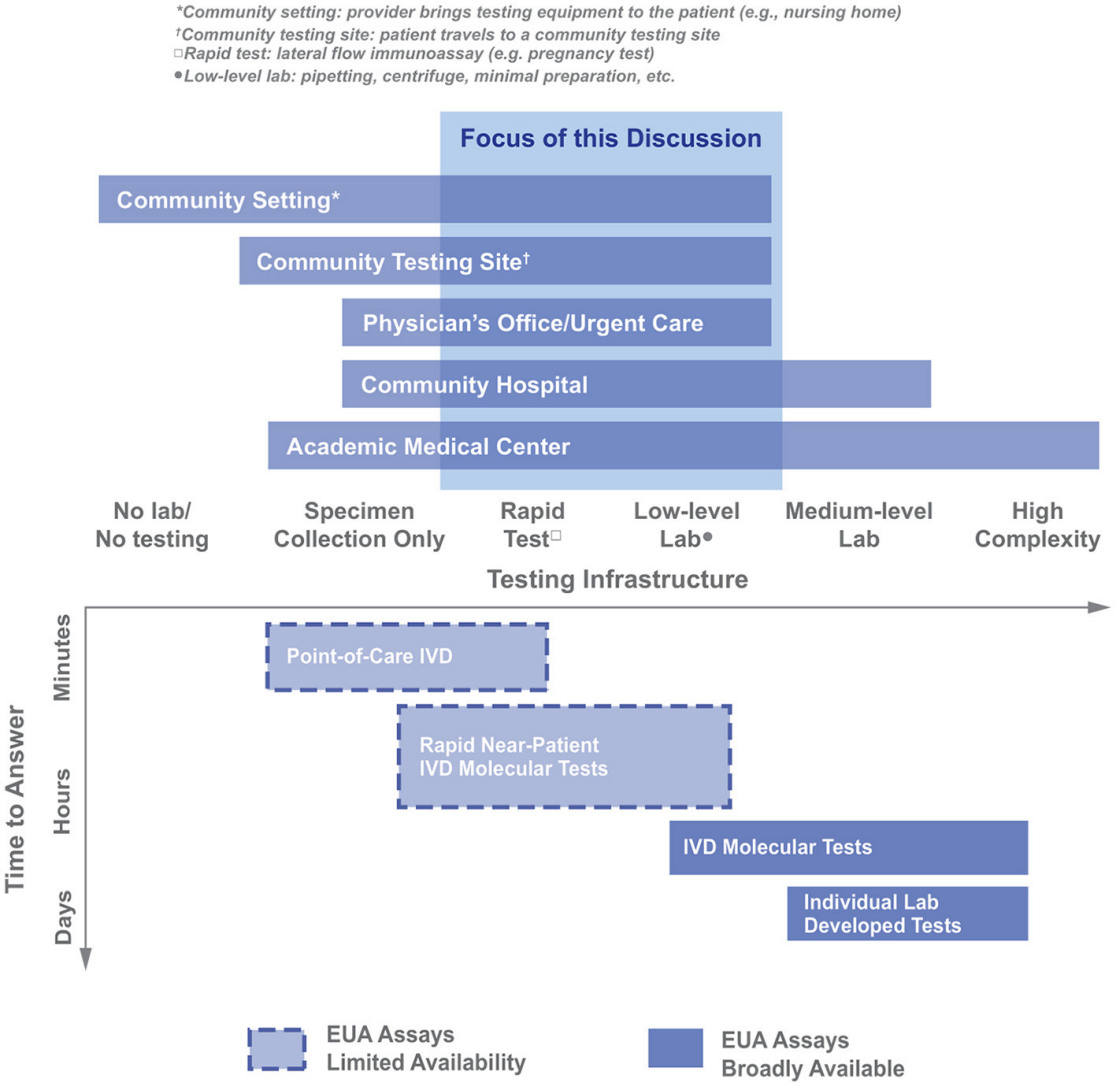
Organizing different healthcare provider settings as a function of the available testing infrastructure in those settings. The Molecular Diagnostic Trade Space is also graphed along the Testing Infrastructure axis (IVD, *in vitro* diagnostic; figure adapted from https://covidinnovation.partners.org/point-of-service-urgent-care/).

It was apparent that the logistical constraints of the POC environment (e.g., infrastructure and staffing availability) would drive the analysis (Figure 3, X-axis). While there are exceptions to the organization shown above, it was agreed that this analysis described most facilities within the scope and would provide a helpful framework going forward. Figure 3 summarizes the different testing locations that may be needed to deploy COVID-19 diagnostics, highlighting the range of testing infrastructure (e.g., power, controlled environment, and equipment) that may be available. The available testing infrastructure also determined, broadly, which classes of diagnostics may be successfully administered on-site (Figure 3). It should be noted that, during the time of the working group's activity (April-June 2020), there were very few diagnostics with emergency use authorization (EUA) status for SARS-CoV-2 and even fewer that were compatible with use in lower-resource settings (Figure 3). In addition to understanding the resources available, there was a need to understand the relative advantages/drawbacks of different classes of molecular diagnostics. No single diagnostic is perfect in all ways; the group spent a significant fraction of its time discussing what “good enough” could be for different metrics and which diagnostic metrics could be relaxed so that others could be optimized. For example, as shown in Figure 3, if speed (minimal time-to-answer) is a top priority, then POC *in vitro* diagnostics are the most promising category; however, this class of diagnostics had limited EUA assays available.

**Figure 4:**
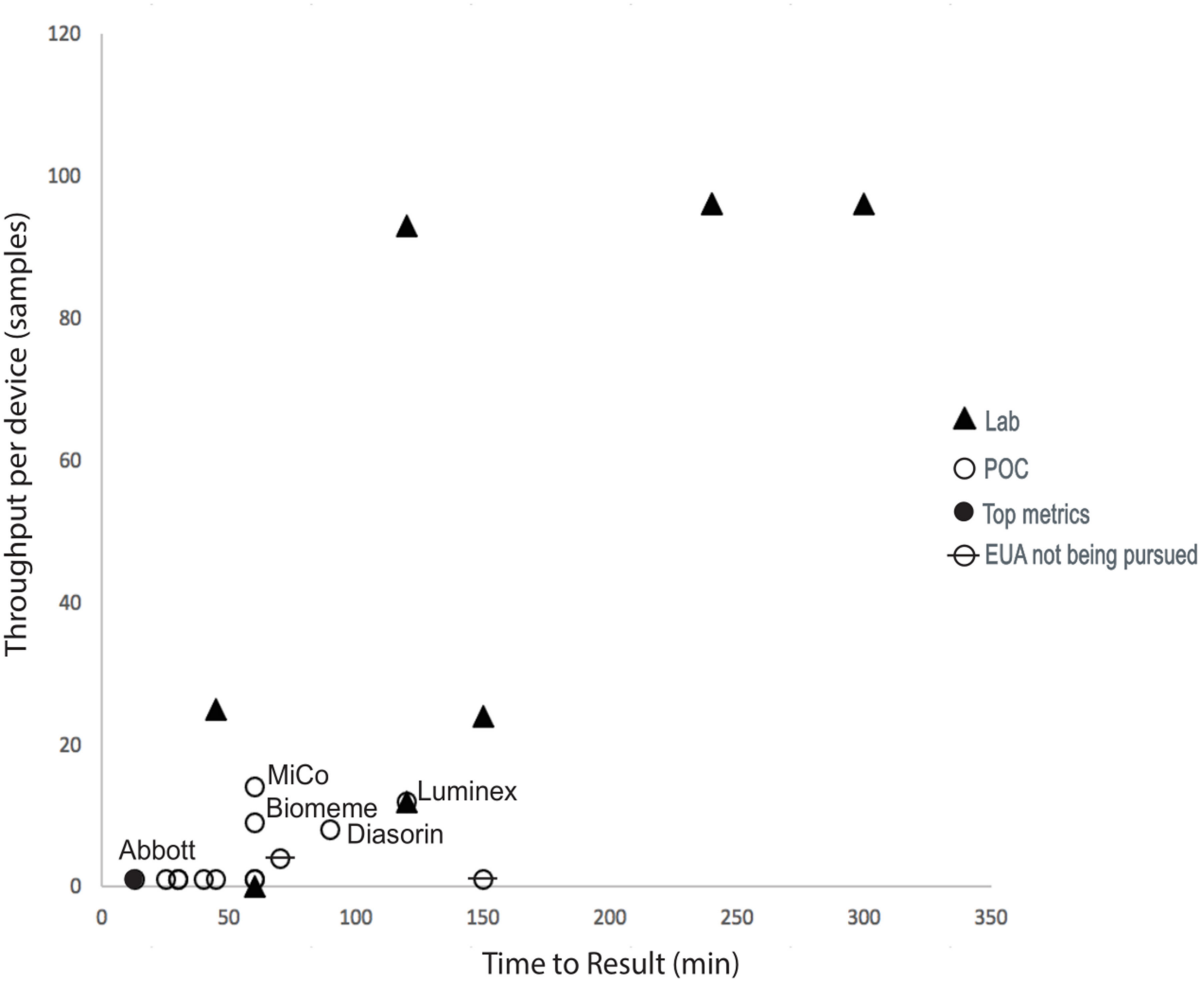
COVID-19 assay landscape during this diagnostics assessment effort. Device throughput and time-to-result for diagnostics available in April 2020. Technologies labeled in the graph were scored with the highest metrics, including supply chain considerations. “Lab” refers to assays that required significant analysis infrastructure (e.g., PCR machines) to be available and were not therefore compatible with operation at point-of-care (POC) settings, which were assumed to have no pre-existing analysis capabilities.

Some aspects of the complex trade space associated with molecular diagnostics and other key metrics (not shown in the figure), such as desired time-to-answer, cost, device throughput, and positive predictive value (PPV)/negative predictive value (NPV), were considered and are captured in the assessment rubric developed during this effort. In April 2020, clear trade-offs existed in the trade space. Figure 4 demonstrates an inverse relationship between throughput and time-to-result POC devices and highlights those assays that were ranked highest when the assessment rubric was applied.

### System Capability Needs

System capability needs refers to the capacity within the hospital or health care infrastructure to adapt and use the diagnostic devices and tests (Walton and Ivers, 2020). Based on the needs of the POC use case, the essential requirements and associated ranking criteria were identified, summarized, and prioritized to determine if the given technologies were well suited to address the operational needs. The critical categories were broadly grouped into two main areas:

(1) Technical: Meet the diagnostic needs. This category initially included several metrics, including limit of detection (LoD), swab type, specificity, and sensitivity. However, as the analysis progressed, it was evident that three main characteristics (“assay type,” “regulatory status,” and “LoD”) were the critical categories:

a. As noted earlier, this category initially included “type of test”—RNA vs. antigen vs. antibody; however, it was decided that, at the time this analysis was being performed, only the assays that were directed toward sensing viral RNA would provide sufficient diagnostic confidence to enable further clinical decision-making.
b. Regulatory status captured the EUA status of each technology.
c. LoD was the sole metric/category that captured assay performance. Please note that the subgroup clearly understood that assay performance is important and that “sensitivity,” “specificity”, and other associated metrics were tracked in the data table. However, for a quick assessment, those metrics were not helpful at the time: for most technologies, it was difficult to obtain this information, and, when reported, the information was rarely reported objectively to allow for ranking or assessment. It was decided that tests that had obtained EUA status would be considered to have adequate performance parameters for this near-term assessment.

(2) Operational: Meet the logistical and supply chain requirements. The assessment began with a high-level understanding of the operational requirements of these settings and as the assessment continued, this understanding was refined and clarified. The logistical and supply chain requirements were further broken down into more specific categories, as described below:

a. Logistics: This set of parameters was most directly impacted by the focus on POC and urgent-care settings. If the operational requirements of those locations were further refined, or if this analysis were applied to other locations, the assessment criteria in these categories would be expected to vary significantly. Within the set of logistics characteristics, the critical categories were as follows:
  i. Assay Complexity: This category was initially defined as “CLIA-waived” but it was evident that, at least during the 2 weeks when this assessment was performed, the FDA was not assessing any assay as CLIA-waived if it included sample collection by a nasopharyngeal (NP) swab. Since during the time of this review all the technologies under consideration did include an NP swab, this category was redefined to capture the minimum necessary lab complexity required to perform a given assay. When available, this information was collected from the FDA EUA approval letter.
  ii. Throughput-per-device and Time-to-Perform: Together, these two parameters combine to provide a first-pass estimate of the number of assays that could be performed per hour/day/shift. Since different testing locations are expected to have various limitations in terms of space, labor, and other resources, it was determined that it would be more useful to separate throughput-per-device and time-to perform as categories for this, and future, assessments.

b. Supply chain: Pragmatically, the best assay in the world is useless if it cannot be reliably obtained. This set of parameters focused on finding quantitative or semi-standardized ways to describe how available and reliable the supply chain was for each technology under evaluation.
  i. Vendor: This category was the most subjective and relied upon the expertise of workgroup members in identifying established, reputable vendors. It was assumed that the more familiar the MGB, or broader, medical community was with the vendor, the more likely it was that the vendor was reliable.
  ii. Hardware: This category captures the degree to which the hardware necessary to run a given assay was already available within the MGB community. Several assays under consideration were designed to be compatible with POC devices already commercially available and, of those, some were already in use within the MGB community. It was assumed that the more integrated these hardware platforms were within the MGB community, the more likely they could be readily available for COVID-19 screening.
  iii. Consumables: The criteria for this category were revised several times to reflect updated feedback from different vendors. Ultimately, assays requiring the use of proprietary buffers, reagents, storage media, or swabs were examined critically concerning supply chain robustness, and assays using more widely available consumables with redundant supply chains were viewed favorably.

### Assessing Candidate Technologies

A multi-pronged data collection and assessment process was developed to identify POC tests for further validation. In addition to the existence of supply chain challenges at the time of this effort, it was also clear that the use case for POC tests was rapidly evolving to include more non-traditional settings (e.g., nursing homes, drive-through testing sites, and airports). For more accurate reporting, a data collection and assessment process was developed to be adapted for different use cases by varying the weighting assigned to test characteristics of interest. Existing and emerging technologies of interest were typically those with high sensitivity and specificity. However, consideration was also given to the form factor of instruments, company reputation, throughput, turnaround time, and type of readout. Company reputation measured the credibility of companies based on their prior success in deploying diagnostics, market penetration and obtaining quality system certifications for medical devices, such as International Standards Organization (ISO) certifications. We predicted that these companies would more efficiently repurpose their existing platforms for SARS-CoV-2 diagnosis, especially if their systems had been previously validated for different diagnostic applications. Several technologies that ranked high were not available for several months and were therefore excluded from the analysis. A questionnaire with information about the technology, the parent company, and its manufacturing processes was completed for promising candidate technologies. The information was entered into a shared spreadsheet created for this purpose (Supplementary Figure 1). The POS working group prioritized molecular tests at the time (April 2020), since these technologies were immediately available for deployment to meet the health care need. The group also recognized the need for evaluating and deploying rapid antigen tests, especially for decentralized and home testing. However, the supply chain of rapid tests was severely limited until the end of 2020. We were subsequently able to access some platforms to evaluate in Massachusetts (Suliman et al., 2021), and in collaboration with global partners who relied more on rapid tests to expand decentralized testing in resource-constrained settings (Kawser et al., 2022; Muthamia et al., 2022). These rapid tests necessitate different parameters in our evaluation rubric since they are known to have lower sensitivities than molecular tests, but can be powerful tools for screening highly infectious individuals with high SARS-CoV-2 viral loads (Guglielmi, 2020; Ricco et al., 2022).

If a large amount of information was missing from publicly available sources, companies were contacted *via* phone or email for additional information. Initial discussions with companies closely followed the questionnaire, and further follow-up was conducted in the case of particularly promising technologies. Working group members were briefed on appropriate questions and how to proceed with obtaining sample assays or additional information *via* a formal agreement, if applicable.

### Scoring Technologies With a Rubric

As mentioned earlier, there are numerous criteria to be assessed when determining which assay system (reagents + hardware) is well-suited for a specific use case. A rubric/assessment metric system was used to assess the suitability of candidate technologies for use in POC/urgent-care settings. Documenting these decision metrics clearly and systematically facilitating discussion helped in reaching consensus. Using terminology and criteria already part of the systems needs assessment and technology assessment simplified the use of this rubric to assess technologies and drive recommendations.

At the time of this assessment, new molecular diagnostics were being announced weekly, if not daily. Top-tier criteria were identified and used as a first-cut of candidate technologies to a short-list of most promising candidates to efficiently manage limited resources and accelerate the timeline to finalize recommendations. These criteria related to pragmatic considerations of regulatory status, the possibility of acquisition, and compatibility with resources available at POS locations, including pharmacies, ambulatory services and urgent care settings.

**Figure 5:**
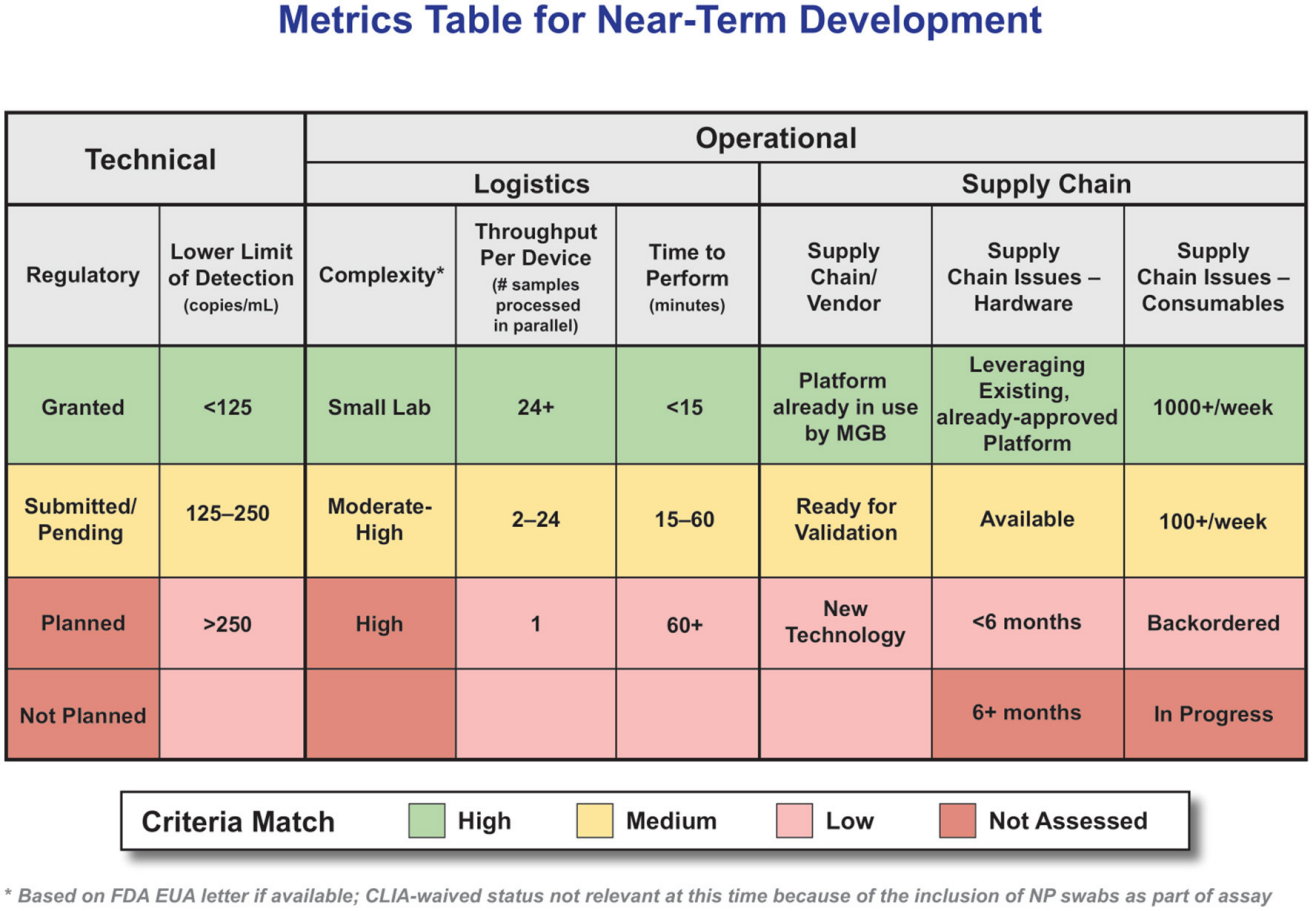
Attributes and Ranking System for POC Molecular Diagnostics. Metrics Table for Near-Term Development. (Table intended to serve as an example, criteria weighting should be adjusted to the specific use-case as needed).

First and foremost was emergency use authorization (EUA) status; only technologies that had submitted an EUA were considered for further assessment; while only those diagnostics that had obtained an EUA could be administered, those that had at least submitted an EUA were still kept in the appraisal because, at the time of this assessment, EUA determinations for diagnostics were progressing rapidly. It seemed possible that technologies could shift from “submitted” to “approved” within a reasonable time frame. The prioritization of key metrics was also strongly informed by the technology assessment; as it became clear that a challenging supply chain was a common concern, the metrics for high/medium/low were modified, and it became a top-tier metric (no matter how otherwise perfect a technology option might be, if it cannot be purchased, it is not helpful). The final top-tier criterion was complexity. Given that the goal was identifying diagnostics for POC use, technology had to be usable (and approved by FDA) in a setting other than a high-complexity laboratory which is not available in most POC use cases such as Urgent Care settings. While initially this criterion was assessed based on CLIA-waived status, that had to be adjusted since, at the time of this assessment, all molecular assays required a nasopharyngeal (NP) swab and could not, therefore, be designated as CLIA-waived. Instead, we deferred to the subject matter experts on the assessment team to provide a subjective assessment of the relative complexity of the laboratory requirements necessary to a given diagnostic. We defined “complexity level” as the additional reagents and equipment needed outside the supplied system to complete the test, e.g., heat blocks and vortexes. Increasing system complexity would increase the reliance on specialized central labs and trained personnel, whereas POS testing aims to simplify and decentralize access to these diagnostics, so they can be used by health care providers outside of specialized clinical microbiology labs, such as in pharmacies, ambulatory services and urgent care settings. Figure 5 highlights the criteria developed to accelerate, analyze, and collect information to focus on high-probability technologies.

Please note there were many other criteria collected for these technologies; this extensive data table remains a valuable resource for more in-depth analysis. One key criterion not shown in the table is cost; while certainly a priority that must be considered, at the time of this assessment there were relatively few technologies that passed the top-tier criteria and those that did were in very high demand. In situations other than a global, rapidly progressing pandemic, it is expected that cost would become a higher priority.

If a technology did not meet all these criteria at any level, it was not assessed further. Still, it did remain on a watch list so that it could be re-assessed if the criteria changed or the technology characteristics changed. It is also important to emphasize that the rubric system is adaptable to meet the testing demands in different contexts. For instance, we can adjust the rubric to assign a higher importance to low cost, and low complexity in rural resource-constrained settings, which face additional challenges (Naidoo et al., 2022).

### Evaluation of Diagnostic Technologies

With the influx of POC technologies to diagnose SARS-CoV-2 infections during the COVID-19 pandemic, including those described in this paper, rigorous criteria to independently evaluate the accuracy and usability of these tests are crucial. Many of these POC tests (e.g., the Accula SARS-CoV-2 test from Mesa Biotech Inc. and the BD Veritor System for Rapid Detection of SARS-CoV-2 from BD Biosciences) obtained EUAs under the condition that they would be used in regulated settings by certified personnel in moderate to high complexity testing labs with CLIA compliance, and would need to show a Certificate of Waiver, Certificate of Compliance, or Certificate of Accreditation, which would allow their use in some but not all health care settings. Ideally, POC tests could include non-accredited technologies that can also be used outside CLIA-compliant settings. This aspect is essential for mass screening and triaging infected individuals in the community during a pandemic. However, decentralized administration of POC tests raised concerns about the accuracy of these platforms and subsequent interpretation of test results by both providers and end-users (Syal, 2021). The pandemic necessitated expedited approvals of diagnostics by the FDA through the EUA process. Thus, the data used to obtain EUA were generally based on small and restricted sample sizes that are often not reflective of the entire population, particularly asymptomatic SARS-CoV-2 infected carriers (Oran and Topol, 2020; Pray et al., 2021; Suliman et al., 2021). Therefore, it is critical that a standardized and rigorous evaluation of the performance characteristics of these diagnostic tests be performed on samples from diverse sources, preferably by third parties with no conflicts of interest regarding the outcome of these evaluations, who can objectively recommend tests for implementation.

Our rubric system highlighted both established manufacturers (e.g., Abbott ID Now™), and new technologies from startup companies (e.g., Fluxergy CoVID-19 Sample-to-Answer RT-PCR). Both technologies have successfully progressed in the diagnostic market, where Abbott ID Now™ has been deployed as a primary diagnostic tool by the US government, and several health care centers, with a pooled sensitivity of Abbott ID Now™ was shown to be 0.79 (95% CI, 0.69–0.86) and 1.00 (95% CI, 0.98–1.00) (Lee and Song, 2021). On the other hand, the Fluxergy COVID-19 Sample-to-Answer RT-PCR have filled a different niche, where it was used in the USA outside of health care settings, in a pooled testing back-to-work application (Rawlings et al., 2021). The company successfully obtained a CE mark, which allows for its deployment in the European market.

## Discussion

The emergence of SARS-CoV-2 variants harboring mutations will directly impact the performance of several diagnostics. If mutations in the SARS-CoV-2 genome impact primer binding sites for molecular tests, the rates of amplification drop-outs will increase, thereby decreasing the sensitivity of these tests. In addition, coding mutations that result in amino acid substitutions may impact the performance of rapid antigen tests that rely on antibodies that recognize the intact viral protein antigens. Therefore, evaluation frameworks that enable rapid evaluation of the performance characteristics of molecular and rapid antigen tests against SARS-CoV-2 variants remain critical.

The shifting landscape of the COVID-19 pandemic challenges our ability to define priorities for validating diagnostic platforms, as newer platforms and technologies are continually developed, rendering former ones obsolete. For instance, more sensitive rapid antigen tests may soon replace PCR platforms for certain applications such as mass surveillance of students and workers currently taking place in many college campuses and organizations, where the goal is to identify infectious individuals, not necessarily everyone who is infected (see, for example, Larremore et al., 2021). Furthermore, access to tests with dwindling supply chain availability and prioritization of tests for immediate implementation has limited test availability for third-party researchers to conduct thorough evaluations. Earlier in the summer of 2020, the US Department of Health and Human Services (HHS) bought millions of rapid antigen tests from Becton Dickinson (BD Veritor) (Young et al., 2020; Kilic et al., 2021; Muthamia et al., 2021), Quidel (Sofia2) (Pray et al., 2021; Smith et al., 2021) and more recently, Abbott (BinaxNOW) (Okoye et al., 2021; Pilarowski et al., 2021; Pollock et al., 2021) as soon as they received EUAs based on limited samples of symptomatic individuals (HHS.gov, 2020a,b). Furthermore, the rapid changes in approval status of tests and the shifting political appetite for different testing modalities meant that the FDA priorities had to accommodate these changes accordingly. To this effect, we intend to maintain a flexible and adaptable pipeline to accommodate evaluations of different types of platforms and technologies as they arise.

The work summarized in this paper was conducted early in the pandemic and focused on assessing diagnostics that could identify if an individual was infected with SARS-CoV-2. As the pandemic enters its third year, other applications for diagnostics, such as “is this individual infectious?” or “is this individual susceptible to SARS-CoV-2 infection (or re-infection)?”, are increasingly important but remain largely unaddressed. Debates regarding the use of rapid tests (see, for example, Guglielmi, 2021) are part of a growing awareness that tests that assess, in an individual, the presence of a pathogen, pathogen component, or evidence of prior pathogen exposure, have a broader scope of use than solely informing subsequent medical decisions for that individual. These other applications, such as informing return-to-work status, may impose a different set of requirements than the more traditional diagnostics applications that are the focus of this paper. The analysis framework presented here can still be applied to facilitate discussion and consensus-building, derive appropriate requirements and prioritization, and assess available technologies against those requirements.

## Data Availability Statement

The original contributions presented in the study are included in the article/Supplementary Material, further inquiries can be directed to the corresponding author.

## Author Contributions

All authors listed have made a substantial, direct, and intellectual contribution to the work and approved it for publication.

## Funding

DRW was funded by the Massachusetts Consortium on Pathogen Readiness (MassCPR) and SS was supported by an award from the Massachusetts Life Sciences Center, Accelerating Coronavirus Testing Solutions (A.C.T.S).

## Conflict of Interest

GM was employed by Mass General Brigham Incorporated. The remaining authors declare that the research was conducted in the absence of any commercial or financial relationships that could be construed as a potential conflict of interest.

## Publisher's Note

All claims expressed in this article are solely those of the authors and do not necessarily represent those of their affiliated organizations, or those of the publisher, the editors and the reviewers. Any product that may be evaluated in this article, or claim that may be made by its manufacturer, is not guaranteed or endorsed by the publisher.
