# Supplementary material for: Systematic Approach to Address Early Pandemic's Diagnostic Unmet Needs
Source: Front Microbiol. 2022 Jun 17;13:910156. doi: 10.3389/fmicb.2022.910156 (PMC9247567; doi:10.3389/fmicb.2022.910156)
Supplement: Supplementary file 1 [file Data_Sheet_1.docx]

**Supplemental Materials**


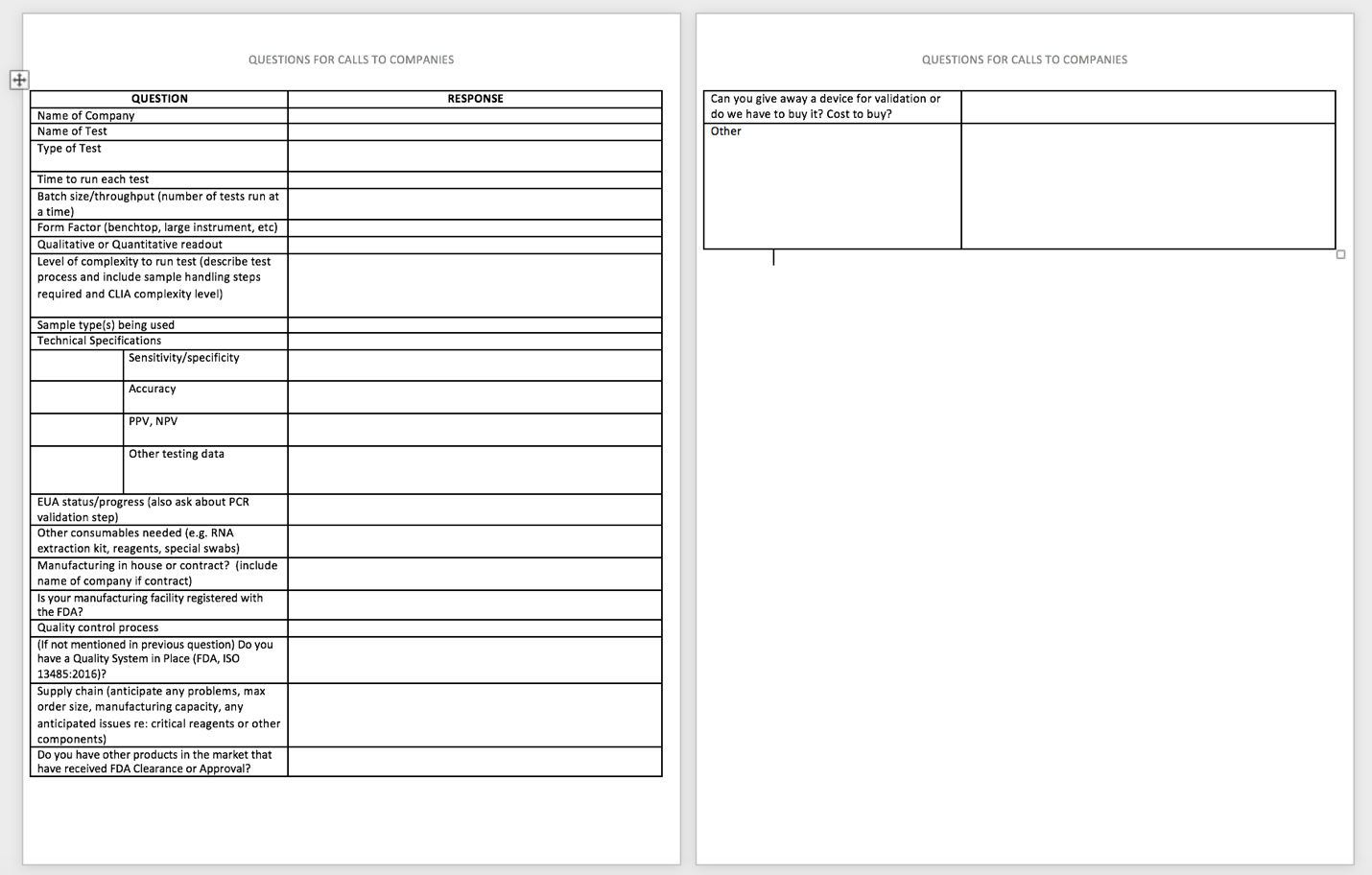


**Supplemental Figure 1:** Questionnaire developed for initial assessment

**Supplemental Table 1:** Table of technologies available in April 2020 separated by target type
